# Assessment of Eicosapentaenoic Acid (EPA) Production from Filamentous Microalga *Tribonema aequale*: From Laboratory to Pilot-Scale Study

**DOI:** 10.3390/md20060343

**Published:** 2022-05-24

**Authors:** Jijian Long, Jing Jia, Yingchun Gong, Danxiang Han, Qiang Hu

**Affiliations:** 1 Center for Microalgal Biofuels and Biotechnology, Institute of Hydrobiology, Chinese Academy of Sciences, Wuhan 430072, China; longjj@ihb.ac.cn (J.L.); springgong@ihb.ac.cn (Y.G.); danxianghan@ihb.ac.cn (D.H.); 2 University of Chinese Academy of Sciences, Beijing 100049, China; 3 Microalgal Biotechnology Center, State Investment and Development Corporation, Beijing 065200, China; jiajing@sdic.com.cn; 4 Institute for Advanced Study, Shenzhen University, Shenzhen 518060, China

**Keywords:** *Tribonema aequale*, EPA, PLA, open raceway pond, microalgal mass culture

## Abstract

It has long been explored to use EPA-rich unicellular microalgae as a fish oil alternative for production of the high-value omega-3 fatty acid eicosapentaenoic acid (EPA, 20:5, n-3). However, none of the efforts have ever reached commercial success. This study reported a filamentous yellow-green microalga *Tribonema aequale* that possesses the ability to grow rapidly and synthesize significant amounts of EPA. A series of studies were conducted in a glass column photobioreactor under laboratory culture conditions and in pilot-scale open raceway ponds outdoors. The emphasis was placed on the specific nutrient requirements and the key operational parameters in raceway ponds such as culture depth and mixing regimes. When optimized, *T. aequale* cells contained 2.9% of EPA (*w*/*w*) and reached a very high biomass concentration of 9.8 g L^−1^ in the glass column photobioreactor. The cellular EPA content was increased further to 3.5% and the areal biomass and EPA productivities of 16.2 g m^−2^ d^−1^ and 542.5 mg m^−2^ d^−1^, respectively, were obtained from the outdoor pilot-scale open raceway ponds, which were the record high figures reported thus far from microalgae-based EPA production. It was also observed that *T. aequale* was highly resistant to microbial contamination and easy for harvesting and dewatering, which provide two additional competitive advantages of this filamentous microalga over the unicellular counterparts for potential commercial production of EPA and other derived co-products.

## 1. Introduction

Palmitoleic acid (PLA, C16:1) is a ω-7 fatty acid that may play a role in preventing chronic metabolic diseases such as insulin resistance, nonalcoholic fatty acid liver disease (NAFLD), obesity, coronary heart disease, and atherosclerosis [1]. Eicosapentaenoic acid (EPA, C20:5) is a ω-3 long-chain polyunsaturated fatty acid (LC-PUFA) that is a constituent of the cell membrane and possesses anticancer and cardio-protective properties [2]. Fish oil is a major source of EPA, but it is facing many issues such as dependency on the food chain, declined fish stock, seasonal supply fluctuations, unpleasant odor and smell, contamination of pesticides and heavy metals, and is unsuitable for vegetarians. Some bioengineered higher plants can also synthesize EPA, but the content is very low, such as transgenic *Arabidopsis thaliana* and *Camelina sativa* seed oil which contain 3.0% and 3.3% of EPA in their fatty acid profiles [3,4]. Conversely, as the most abundant primary producer in the aquatic environment, many microalgae can convert solar energy and carbon dioxide (CO_2_) into desirable products including PLA and/or EPA. For example, *Isochrysis galbana* [5], *Thalassiosira pseudonana* [6], *Nannochloropsis* spp. [7], *Phaeodactylum tricornutum* [8], *Halamphora coffeaeformis* [9], *Tetraselmis* spp. [10], *Nitzschia laevis* [11], and *Monodus subterraneus* [12] produce EPA, whereas *Scenedesmus obliquus* [13], *Eustigmatos* spp. [14], and some cyanobacterial species [15] produce PLA. However, large-scale cultivation of these unicellular microalgae often encounters two difficulties. The first difficulty is contamination of microalgal culture with predatory protozoa and zooplankton or pathogenic bacteria and fungi, often resulting in considerable reduction in biomass productivity or culture crash, losing the culture altogether [16,17]. The second one is the high cost associated with harvesting of these unicellular microalgal cells by centrifugation. As a result, mass cultivation of these unicellular microalgae has not been commercially successful.

In recent years, the freshwater filamentous microalgae *Tribonema* spp., in the class *Xanthophyceae*, have drawn great attention because they possess the ability to produce both PLA and EPA, are resistant to grazers or predators, and have low cell harvesting costs owing to the filamentous nature of these species [18]. As far as fatty acids are concerned, most studies on *Tribonema* spp. have so far devoted to PLA production in an autotrophic or a heterotrophic culture mode, and little attention was paid to EPA [19,20]. It was widely reported that decreased macronutrients of nitrogen and phosphate levels exerted considerable effects on EPA biosynthesis and distribution in other EPA-producing microalgae species [21,22]. Metal ions in the medium are essential substances that play crucial roles in the physiological and metabolic processes of algae. In particular, magnesium occupies a central position in chlorophyll molecules [23]. If allowed for the connection between PUFA enrichment and chloroplasts [24], magnesium should be considered as one of the factors that can regulate the production of EPA.

Open raceway ponds (ORP) have been used for mass culture of several microalgae of commercial interest, such as *Arthrospira platensis*, *Chlorella* spp., *Dunaliella salina*, and *Haematococcus pluvialis* [25,26,27]. Compared with its counterpart, closed tubular photobioreactors, an ORP offers great advantages of having lower capital and operational costs per unit of the illuminated surface area of photobioreactor or culture volume. However, ORP-based commercial-scale cultivation of EPA- or PLA-producing unicellular microalgae in ORP has not been possible due to the above-mentioned difficulties, particularly associated with the unicellular species of microalgae [28,29]. Thus far, the research on the cultivation of *Tribonema* spp. Are mostly performed at laboratory scale, and a very few attempts were made to grow filamentous *Tribonema* spp. In ORP [30].

To obtain high productivity of microalgal biomass or desirable products in ORP, an appropriate depth of culture suspension is critically important. The areal productivity generally increases with the increase in culture depth. Compared with 20 cm depth ponds, for example, the areal biomass productivity at a depth of 40 cm increased nearly 2-fold [31]. Moreover, one ultra-high depth pond of 1 m was designed to cultivate *Arthrospira platensis*, which achieved areal productivity of 21.22 g m^−2^ d^−1^, while another conventional pond with a lower depth only had a productivity of 11.05 g m^−2^ d^−1^ [32]. On the contrary, some researchers did not find that increasing culture depth can significantly improve the areal productivity. For example, areal productivity for *Tetraselmis suecica* was 8.37 g m^−2^ d^−1^ in 15 cm depth ORP, which was comparable with 8.9 g m^−2^ d^−1^ in 5 cm depth ORP [33].

Different culture mixing regimes, namely continuous culture mixing versus culture mixing provided only during the daylight period, may not only affect energy consumption associated with the operation of the paddlewheel but also influence the occurrence and development of predatory protozoa and zooplankton that prey on microalgae. It was reported that no difference in *Chlorella* biomass productivity was observed for both continuous and daytime-only mixing [34]. However, other studies indicated that continuous mixing was better than daytime mixing, likely due to favorable pH gradient and exchange of gases [35,36].

A series of exploratory studies are needed for a potential EPA-producing algal strain, thus the present work aimed at the maximization of EPA production by a *Tribonema* strain grown under both laboratory and outdoor open conditions with different reactors or scales. For this purpose, optimization of the nutrient requirement for algal growth is also a part of this work. In batch culture, the algal growth and EPA production are also monitored as a part of this work.

## 2. Results

### 2.1. Effect of Excessive Phosphate and Magnesium Sulphate on the Growth of T. aequale SAG200.80

Zarrouk’s medium was the synthetic culture medium originally formulated for the cultivation of *Spirulina* spp. [37], which contains high concentrations of nutrients, particularly NaHCO_3_, NaCl, and K_2_SO_4_, which create a high-salt and high-alkali environment. These three chemicals were discarded from Zarrouk’s medium and named modified Zarrouk’s medium, or M-zarrouk. BG11 medium and M-zarrouk medium, which differs from M-zarrouk medium mainly in phosphorus and magnesium concentrations (0.0524 vs. 0.655 g L^−1^ and 0.075 vs. 0.2 g L^−1^, respectively), were used. Cultivation of *T. aequale* was subsequently carried out in five kinds of culture medium, including BG11, M-Zarrouk, BG11 medium with excess phosphorus (BG11 + P), BG11 medium with excess magnesium (BG11 + Mg), and BG11 medium with excess phosphorus as well as magnesium. CO(NH_2_)_2_, K_2_HPO_4_·3H_2_O, and MgSO_4_·7H_2_O were used as nitrogen, phosphorus, and magnesium sources, respectively. The nutrient composition of each medium was listed in Table 1.

**Figure 1:**
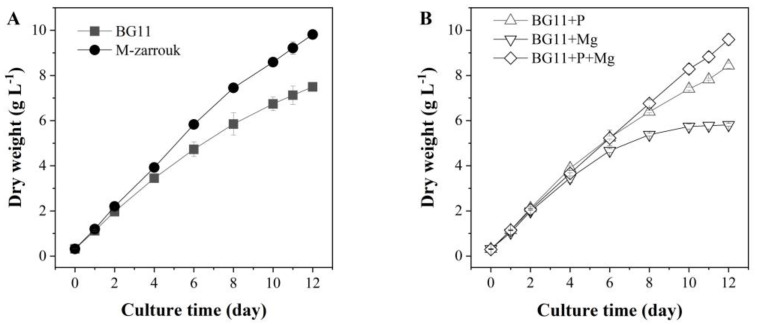
(**A**) Effects of the culture media BG11 and M-Zarrouk on biomass production of *T. aequale*, and (**B**) growth of *T. aequale* as affected by spiking the additional amount of phosphate (0.60 g L^−1^) or magnesium (0.13 g L^−1^) or both in the BG11. Experiments were conducted in glass columns (4.3 cm inner diameter) that each contained 900 mL culture medium. Culture temperature was 25 °C, and cool white fluorescence light was provided continuously at a light intensity of 200 μmol m^−2^ s^−1^. Culture pH was maintained at pH of 7.5–8.0 by providing compressed air bubbles containing 1~2% CO_2_. Values are expressed as mean ± standard deviation of three replicates.

*T. aequale* grew rapidly in BG11 and a biomass concentration of 7.5 g L^−1^ was observed after 12 days of cultivation. However, a 30% increase in biomass yield (9.81 g L^−1^) was obtained in *T. aequale* culture maintained in the M-Zarrouk medium. The addition of 0.2 g L^−1^ MgSO_4_ to the BG11 medium significantly increased biomass yield as compared to that from the BG11 cultures, whereas the addition of 0.655 g L^−1^ K_2_HPO_4_ to the BG11 medium resulted in the reduction in growth. Interestingly, when the BG11 medium was spiked with both 0.2 g L^−1^ MgSO_4_ and 0.655 g L^−1^ K_2_HPO_4_, the final biomass yield of *T. aequale* cultures was as high as that with the M-Zarrouk medium (Figure 1).

**Figure 2:**
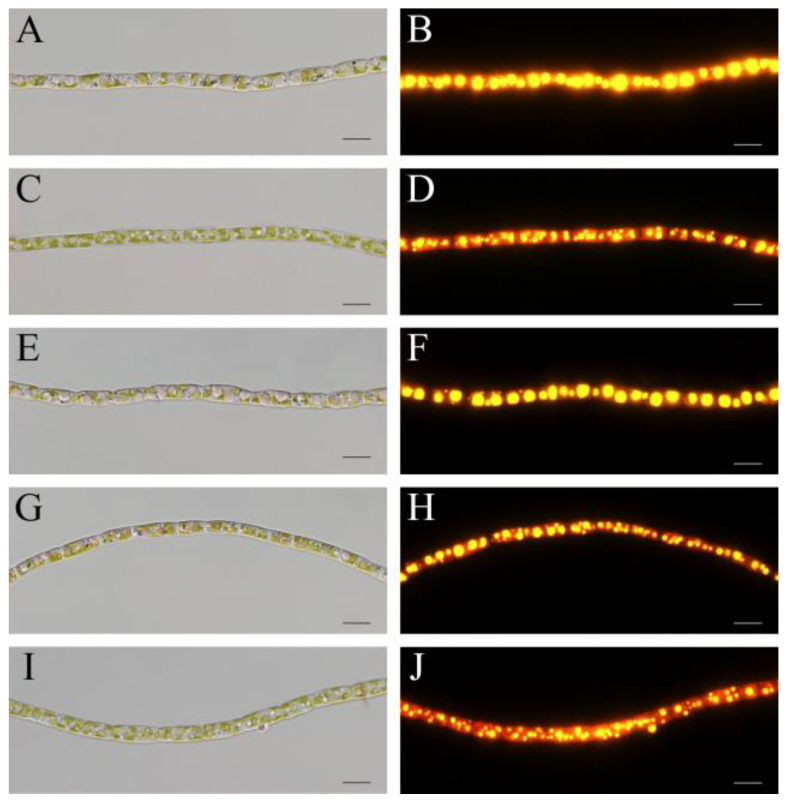
Morphological observations of *T. aequale* under bright field (**left**) and fluorescence microscopes (**right**). *T. aequale* was cultured in BG11 (**A**,**B**), BG11 + P (**C**,**D**), BG11 + Mg (**E**,**F**), BG11 + P + Mg (**G**,**H**), and M-zarrouk (**I**,**J**) and samples were taken on day 12 for microscopy. Cellular lipid bodies were stained with the fluorescent dye Nile Red. Scale bars, 10 μm.

The cell morphology and intracellular lipid bodies were observed with normal optical and fluorescence microscopy. While the cell size of *T. aequale* filaments was more or less the same, the number and size of intracellular lipid bodies were noticeably different under the different culture conditions. Large lipid bodies were evident in *T. aequale* grown in the BG11 medium, whereas much smaller lipid bodies occurred in the *T. aequale* cells maintained in the M-Zarrouk medium. The appearance of lipid bodies in the BG11 + P cultures was similar to that in the BG11 medium, whereas greater numbers but smaller sizes of lipid bodies were observed in the cells grown in either the BG11 + Mg or the BG11 + P + Mg (Figure 2).

**Figure 3:**
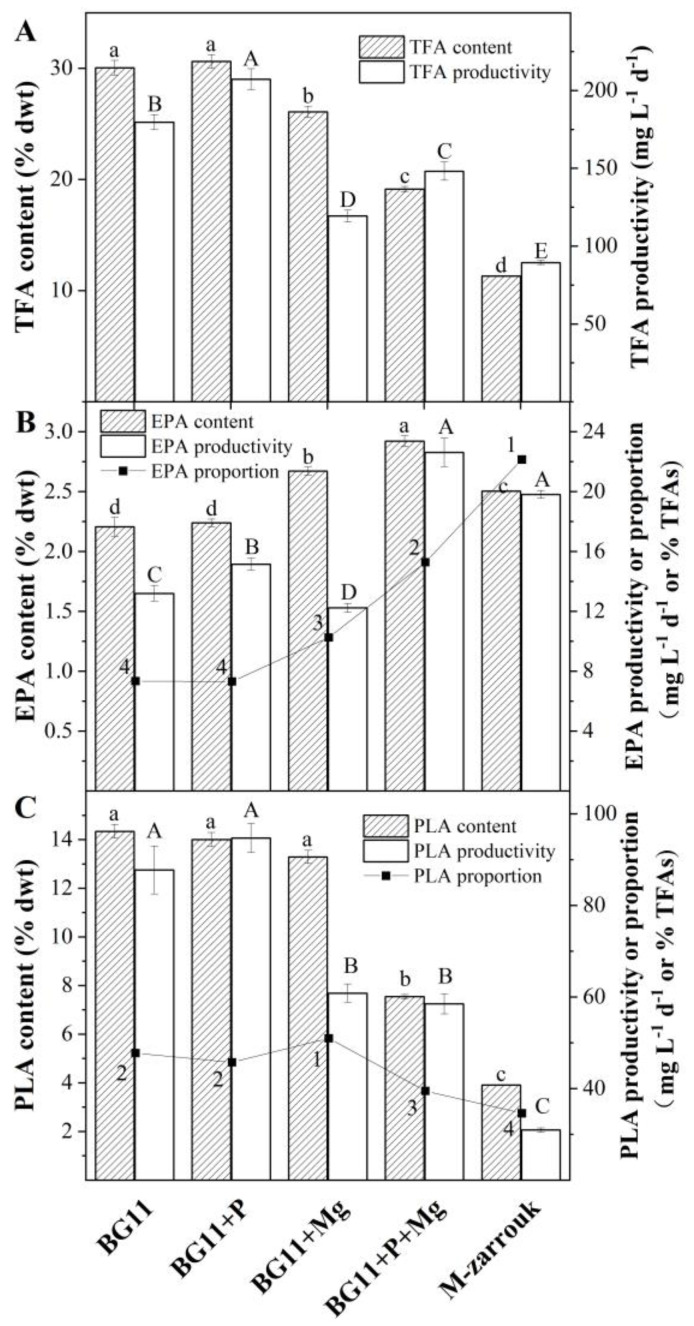
The cellular contents of TFA, EPA, and PLA, proportions of EPA and PLA in TFA, and productivities of TFA, EPA, and PLA of *T. aequale* grown in glass columns containing the different culture media: BG11, BG11 + P, BG11 + Mg, BG11 + P + Mg, and M-Zarrouk ((**A**): TFA; (**B**): EPA; (**C**): PLA). The values from the experiments (*n* = 3) are shown as mean ± one standard deviation. Different lowercase letters, capital letters, and Arabic numerals indicate significant differences among the EPA, PLA, or TFA contents, EPA, PLA, or TFA productivity, and EPA or PLA proportion in TFAs, respectively.

The total fatty acid (TFA) content of *T. aequale* was 30 ± 0.6% of cell dry weight (DW) after 12 days of cultivation in BG11 and BG11 spiked with 0.6 g L^−1^ K_2_HPO_4_ (BG11 + P). It decreased to 26 ± 0.5%, 18 ± 0.2%, and 12 ± 0.1% in BG11 + Mg, BG11 + P + Mg, and M-Zarrouk, respectively. As the productivity of TFA is a function of cellular TFA content and biomass concentration, the maximum TFA productivity of 200 mg L^−1^ d^−1^ was obtained from the BG11 + P cultures. A slightly lower TFA productivity (180 mg L^−1^ d^−1^) was observed in the BG11 cultures, but much lower TFA productivities of 120, 150, and 90 mg L^−1^ d^−1^ were measured in BG11 + Mg, BG11 + P + Mg, and M-Zarrouk cultures, respectively (Figure 3A).

However, the cellular content of EPA, proportion of EPA in TFA, and productivity of EPA in *T. aequale* grown in the different culture media exhibited quite different trends from that of TFA. The highest EPA content (2.92%, *w*/*w*) and the proportion of EPA (15.3%) in TFA were obtained in the BG11 + P + Mg cultures, followed by the M-Zarrouk and BG11 + Mg cultures. The lowest EPA content and proportion of EPA in TFA occurred in the BG11 and BG11 + P cultures. As a result, the highest EPA productivity of 22.63 mg L^−1^ d^−1^ was obtained in the BG11 + P + Mg cultures (Figure 3B).

The cellular content of PLA, proportion of PLA in TFA, and productivity of PLA followed essentially the same trends as that of TFA. A high PLA content of 48% in TFA and the highest PLA productivity of 94.7 mg L^−1^ d^−1^ were achieved in both BG11 and BG11 + P cultures (Figure 3C).

### 2.2. Outdoor Cultivation in S-ORPs

#### 2.2.1. The Effects of Different Culture Media on the Production of Biomass, EPA, and PLA

To move one step further, *T. aequale* was tested in ORP outdoors using three different culture media (BG11, 1/2BG11, and BG11 + P + Mg) for the production of biomass, EPA, and PLA. Each ORP had a culture surface area of 0.56 m^2^ and a culture volume of 133 L. The only difference between 1/2BG11 and BG11 was that the former contained a half of the amount of CO(NH_4_)_2_ used in the BG11. It revealed that the alga grew well in these media, resulting in more or less the same final biomass concentration of 1.1 ± 0.03 g L^−1^ and biomass productivity of 15.5 ± 0.73 g m^−2^ d^−1^. However, the EPA and PLA contents in the algal cells grown in 1/2BG11 were significantly higher than that in the BG11 and BG11 + P + Mg cultures, resulting in the highest productivities of EPA (542.5 ± 27.1 mg L^−1^ d^−1^) and PLA (570.4 ± 34.6 mg L^−1^ d^−1^) (Table 2).

The fatty acid profiles of *T. aequale* cultivated in ORP with the three different culture media were further analyzed and the results are shown in Table 3. The fatty acids were classified into saturated FAs (SFA), monounsaturated FAs (MUFA), and polyunsaturated FAs (PUFA). The most abundant FAs were PUFAs, which accounted for 51.25 ± 0.68% of TFAs, followed by MUFAs (33.25 ± 0.10%, TFA). SFAs represented the least amounts of TFAs (15.49 ± 0.03%, TFA). PLA and EPA were the most abundant fatty acids, making up 31.47 to 32.87% and 29.55 to 31.26% of TFA, respectively.

#### 2.2.2. The Effects of Culture Depth of S-ORP on the Production of Biomass, EPA, and PLA

**Figure 4:**
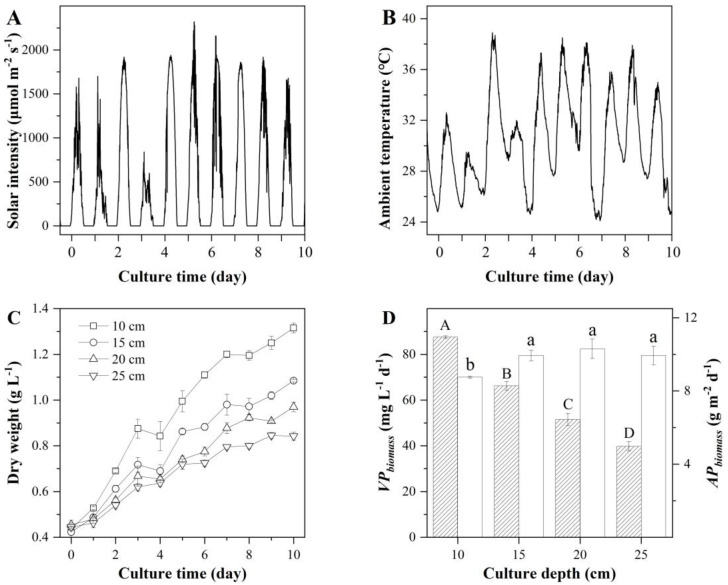
Solar intensity (**A**), ambient temperature (**B**), biomass concentration (**C**), and volumetric (filled column) and areal (blank column) biomass productivities of *T. aequale* (**D**) maintained in 0.56 m^2^ S-ORP outdoors at the culture depths of 10, 15, 20, and 25 cm. Different capital letters and lowercase letters indicate significant differences among volumetric and areal biomass productivities, respectively. The experiment was carried out from 18–27 July 2019.

To further increase the productivity of EPA and PLA, the culture depth in the S-ORP was optimized. The assessment was made with 1/2BG11 culture medium in a batch culture mode. Four culture depths were examined, i.e., 10, 15, 20, and 25 cm. The solar irradiances and ambient temperatures during the experimental period are shown in Figure 4. An average photon flux density during the daylight period was 841.85 μmol m^−2^ s^−1^ with a maximum photon flux density of 2320 μmol m^−2^ s^−1^ (Figure 4A). The culture temperatures in the S-ORP of various culture depths are shown in Figure 4B. The culture pH was manually controlled by bubbling the culture with a stream of pure CO_2_ during the daylight period.

The alga grew well in the S-ORP of various culture depths and yet the shallower the culture depth the more rapid growth occurred on a per-volume basis. The final biomass concentrations were 1.32, 1.09, 0.97, and 0.84 g L^−1^ in the 10, 15, 20, and 25 cm deep S-ORP, respectively (Figure 4C). When biomass productivities were calculated on a per-illuminated surface area of S-ORP (Figure 4D), however, it turned out that the S-ORPs varying in culture depth from 15 cm to 25 cm resulted in the same areal biomass productivity of 9.94 ± 0.28 g m^−2^ d^−1^, 10.3 ± 0.53 g m^−2^ d^−1^, and 9.94 ± 0.52 g m^−2^ d^−1^, respectively. This was significantly higher than that of 8.76 g m^−2^ d^−1^ (*p* < 0.05) from the 10 cm ORP.

The TFA contents in *T. aequale* grown in the 15, 20, and 25 cm S-ORP for 10 days were 12.27%, 12.53%, and 12.40%, which was slightly higher than that (11.56% DW) achieved in the 10 cm S-ORP. The highest areal TFA productivity of 1291.07 ± 16.96 mg m^−2^ d^−1^ was obtained in the 20 cm S-ORP, which also yielded the highest areal productivities of EPA (348.02 ± 4.93 mg m^−2^ d^−1^) and PLA (449.20 ± 5.19 mg m^−2^ d^−1^) (Table 4).

### 2.3. Effects of Different Culture Mixing Regimes on the Production of Biomass, EPA, and PLA

A comparison was made between the cultures with continuous culture mixing and the ones with mixing occurred only during the daylight period with regard to the production of algal biomass, EPA, and PLA.

**Figure 5:**
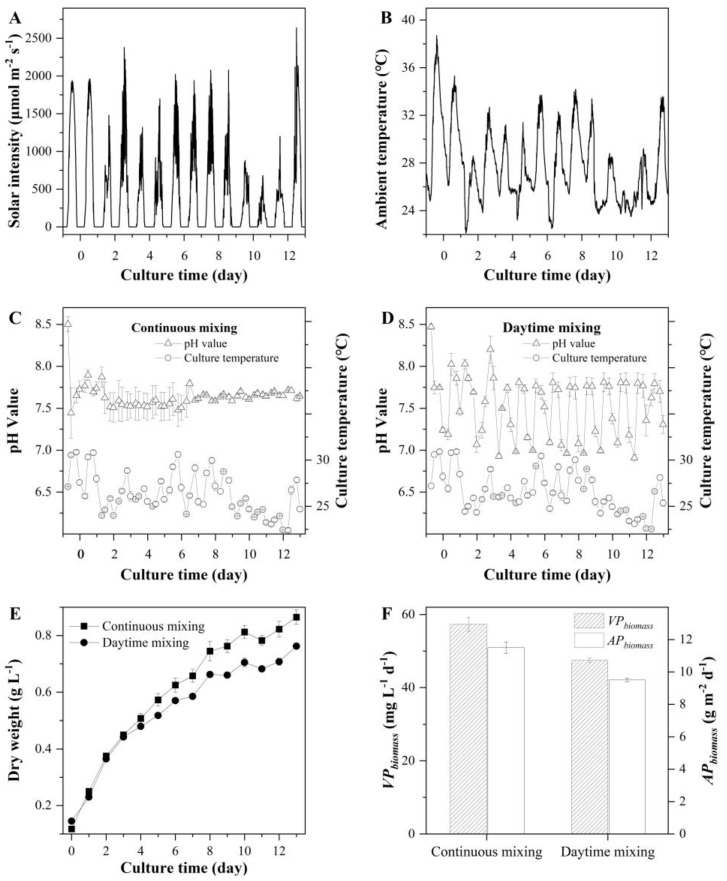
Solar intensity (**A**), ambient temperature (**B**), pH values and culture temperature (**C**,**D**), biomass concentration (**E**), and volumetric and areal biomass productivities (**F**) of *T. aequale* grown in 5.2 m^2^ M-ORP operated in a continuous mixing regime and a daytime mixing one. Each treatment had two biological replicates. The experiment was carried out from 31 July to 13 August 2019.

The experiment was carried out in M-ORP (illuminated surface area of 5.2 m^2^) under outdoor environmental conditions. It revealed that (Figure 5) *T. aequale* grew gradually with both mixing regimes, and the final biomass concentrations of the cultures with continuous mixing and daytime mixing pond culture were 0.87 ± 0.03 g L^−1^ and 0.76 ± 0.01 g L^−1^, respectively. The volumetric and areal biomass productivity in the continuous mixing ponds were 57.3 mg L^−1^ d^−1^ and 11.5 g m^−2^ d^−1^, respectively, which were about 20% higher than that from the cultures with mixing operated only during the daylight period (Figure 5F). It was also determined that the cultures with the continuous mixing regime had the cellular EPA, PLA, and TFA contents of 3.23% (*w*/*w*), 4.50% (*w*/*w*), and 11.89% (*w*/*w*), respectively, which were 6.95%, 18.11%, and 11.64% higher than those obtained with the periodic mixing regime (Table 5).

### 2.4. Demonstration of the Optimized Culture Protocol for Mass Culture of T. aequale in a Large-ORP (L-ORP) Outdoors

**Figure 6:**
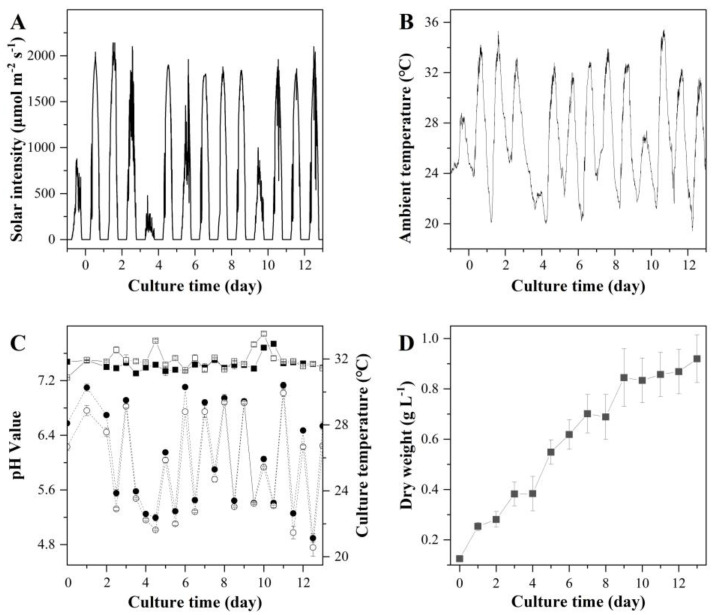
Solar intensity (**A**), ambient temperature (**B**), culture pH and temperature (**C**), and biomass concentration (**D**) of *T. aequale* grown in two outdoor 52 m^2^ L-ORP (L-ORP-1 and L-ORP-2) (filled square, hollow square, filled cycle, and hollow cycle represent pH values of L-ORP-1 and L-ORP-2, culture temperatures of L-ORP-1 and L-ORP-2, respectively). The experiment was carried out from 16–29 August 2019.

Based on the results from S-ORP and M-ORP trials an optimized culture maintenance protocol was established as follows: 1/2BG11 culture medium, 20 cm culture depth, and continuous culture mixing. This protocol was demonstrated in two large-ORP (L-ORP) outdoors. During the cultivation period, the average and maximum solar irradiances were 924.23 μmol m^−2^ s^−1^ and 2000 μmol m^−2^ s^−1^, respectively (Figure 6A), and the lowest, highest, and average ambient temperatures were 24.6 °C, 35.4 °C, and 26.6 °C, respectively (Figure 6B). The variations in pH and culture temperature in L-ORP are shown in Figure 6C. As shown in Figure 6D, *T. aequale* grew steadily and reached the final biomass concentrations of 0.93 ± 0.1 g L^−1^ and the areal biomass productivity of 12.5 ± 1.2 g m^−2^ d^−1^ after 13 days of cultivation. The fatty acid composition and productivities of EPA, PLA, and TFA are summarized in Table 6. It showed that the cellular contents of EPA, PLA, and TFA in the L-ORP were 3.2%, 3.9%, and 11.6% (*w*/*w*), which were more or less the same as those measured in S-ORP and M-ORP (Table 2, Table 4 and Table 5). These results suggested that the optimal culture maintenance protocol developed in this study was accurate and reliable regardless of the size of the ORP.

### 2.5. Microbial Contamination and Potential Impact on T. aequale Cultures

**Figure 7:**
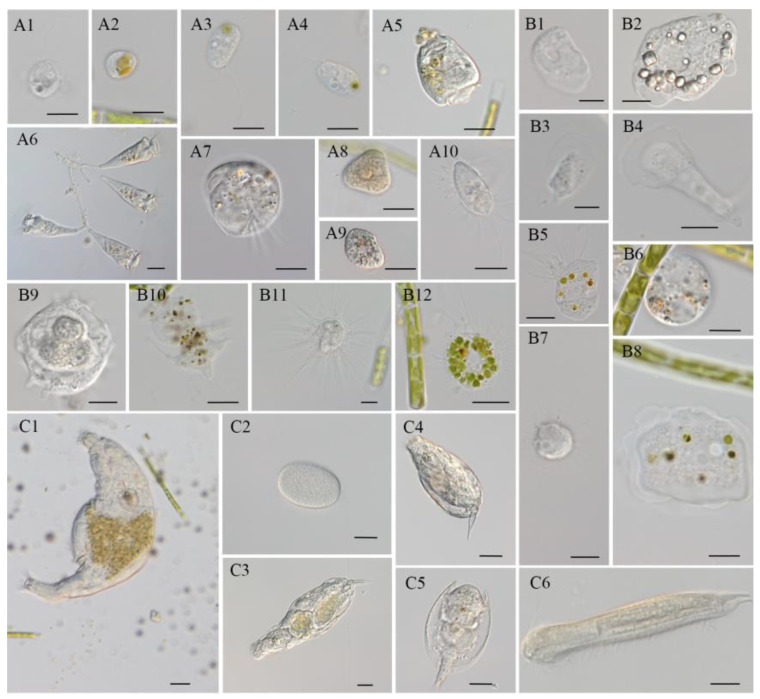
Protozoa and zooplankton occurred in *T. aequale* cultures outdoors, which were classified into three categories: flagellates and ciliates (**A**), amoeba (**B**), and rotifers and metazoans (**C**). (**A1**,**A2**): *Poterioochromonas* sp.; (**A3**,**A4**): flagellates (unknown); (**A5**): *Vorticella convallaria*; (**A6**): *Epistylis* sp.; (**A7**): *Aspidisca* sp.; (**A8**): *Suctorian* sp.; (**A9**): *Colpoda* sp.; (**A10**): *Cyclidium* sp. (scale bar: (**A1**–**A4**, **A7**, and **A10**) = 10 μm; (**A5**, **A6**, **A8**, and **A9**) = 20 μm). (**B1**–**B4**, **B8**, and **B9**): *Vannella* sp.; (**B5** and **B12**): *Nuclearia* sp.; (**B6** and **B10**): unknown amoeba; (**B7** and **B11**): *Heliozoa* sp. (scale bar: (**B1** and **B3**) = 5 μm; (**B2**, **B5**–**B9**, **B11**, and **B12**) = 10 μm; (**B4** and **B11**) = 20 μm); (**C1**): *Philodina* sp.; (**C2**): Rotifer egg; (**C3**): *Lecane inermis*; (**C4**): *Monostyla* sp.; (**C5**): *Lepadella patella*; (**C6**): *Chaetonotus* sp. (scale bar = 20 μm).

Although all the *T. aequale* cultures in this study began with monoalgal inocula without any noticeable contamination by protozoa and zooplanktons, these microorganisms occurred in the cultures in just a few days. Yet, the number and phylogenetic diversity of the contaminated microbial species or strains increased as the cultures were maintained for a longer period. A total of 18 species/strains of microbial contaminants in *T. aequale* cultures were observed under a light microscope, and these microbes were classified into three groups, flagellates/ciliates, amoeba, and rotifers (Figure 7). It was observed that some microbes, such as *Vannella* sp., *Nuclearia* sp., and *Voticella convallaria*, may graze microalgal cells, but the others such as *Poterioochoromonas* sp., *Epistylis* sp., *Helizoa* sp., and *Chaetonotus* sp. never preyed on microalgae. Interestingly, these microbes preyed on unicellular microalgae, but not the filamentous *T. aequale*. Therefore, during our entire experimental period, we did not experience any culture crashes due to microbial contamination.

## 3. Discussion

Microalgae have long been regarded as a natural source of EPA and PLA, and a number of unicellular microalgae (e.g., *Nannochloropsis* spp., *Monodus subterraneus*, *Phaeodactylum tricornutum*, *Eustigmatos vischeri*, and *Nitzschia laevis*) largely from Bacillariophyceae and Eustigamtophyceae have been subjected to investigation. However, none of these unicellular microalgae with adopted mass culture technologies has made commercial success, due mainly to microbial contamination in mass culture of microalgae, resulting in unsustainable cultures with severe reduction in productivity of microalgal biomass or desirable product. Another reason was a projected high cost associated with harvesting and dewatering of the unicellular microalgal cells by centrifugation. It was estimated that harvesting of unicellular microalgae from culture broth may account for 20–30% of the total cost of microalgal biomass production [38].

Most previous research with several *Tribonema* species and strains was focused on PLA production [20,39]. In this study, we reported the new *Tribonema* strain *T. aequale* as a potential EPA producer in addition to the production of PLA. When the culture conditions were optimized, the EPA content in *T. aequale* cells was 3.5%, which made up ca. 27% of total fatty acids in the cell, resulting in an EPA productivity of 542.5 ± 27.1 mg m^−2^ d^−1^. Their results, along with the findings from Davis et al. [30], demonstrated that the cellular content and productivity of EPA from culture of *Tribonema* spp. are comparable to, if not greater than, those from EPA-producing unicellular microalgae grown in raceway ponds outdoors (Table 7). The high EPA productivity obtained from this study was likely due to the filamentous nature of this organism which provides it with high resistance to microbial contamination (such as protozoa and zooplankton) that otherwise can be the most severe threat to the mass culture of unicellular microalgae, in particular in open raceway ponds [40]. It was reported that a fungal parasitoid of algae, an *Aphelidium* strain, can encyst and penetrate *Tribonema gayanum* through an infection tube to engulf the algal cytoplasm [41,42]. However, we did not observe any infection or noticeable negative impact of *Tribonema* cultures by any fungal parasitoid during a year-long study of the mass culture of *T. aequale* in ORP outdoors. We speculated that infection of microalgae by the fungal parasitoid *Aphelidium* might be species-specific, or the environmental and nutrient conditions set for the culture of *T. aequale* in this study did not sustain rapid growth and proliferation of the parasitoid. It seems that the resistance to contamination by protozoa and/or zooplankton is the common feature of those microalgae with a filamentous form, as this phenomenon was also observed in the mass culture of various filamentous microalgae and cyanobacteria, such as another *Tribonema* species *T. minus* [30], the filamentous green microalgae *Klebsormidium* sp. Lgx80 for lipid production [43], *Oedocladium carolinianum* for astaxanthin production [44], and *Arthrospira platensis* for protein production [25]. As some protozoa and zooplankton strains did graze unicellular microalgae (Figure 7(B8,B12)), these grazers may actually protect the filamentous strain *T. aequale* from the invaded unicellular microalgae, and thus make the filamentous microalgal culture more sustainable.

Another added benefit of the filamentous form of *T. aequale* cells is easy and cost-effective harvesting and dewatering. Instead of using a more capital- and operation-intensive centrifugation technique, a proper sedimentation or filtration technique can be readily applied for harvesting and dewatering of filamentous microalgae [45].

Due to the presence of photosynthetic pigments, in particular chlorophylls, in microalgal cells, light impinging on the surface of the culture suspension may be attenuated rapidly, leaving a portion of microalgal cells in the dark at any moment, yet the higher the cell concentration the shallower the culture depth that light may penetrate. Therefore, culture depth is an important parameter in open raceway ponds that may affect not only microalgal growth but also biochemical composition of the cells. It was reported that a culture depth of 12–15 cm was optimal for production of algal storage lipids [46,47] and secondary carotenoids [48]. In this study, however, the optimal culture depth was 20–25 cm for a maximum cellular EPA content of 3.4% (*w*/*w*) and a higher EPA productivity of 344.5 mg m^−2^ d^−1^ than that obtained from the cultures maintained at 10–15 cm depth (i.e., 3.1% EPA and EPA productivity of 290 mg L^−1^ d^−1^) (Table 4). It seems that maintaining a relatively shallow culture suspension in an ORP may enhance production of high light-induced products such as storage neutral lipids and secondary carotenoids [49], whereas a greater culture depth may facilitate formation of low light-enhanced biosynthesis of EPA-containing polar membrane lipids such as phospholipids and glycolipids [7,50,51] and photosynthetic pigments such as phycobiliproteins and fucoxanthin in algal chloroplasts [52,53].

Proper mixing of culture suspension by means of a paddle wheel in an ORP is a prerequisite for improved microalgal photosynthesis and thus biomass productivity. However, it might be case by case whether or not culture mixing would be necessary at night when light is not available. An apparent reason for lowering the mixing rate or stopping mixing at night is to reduce energy consumption and thus operational costs [54]. Another possible positive advantage of halting culture mixing at night is to reduce oxygen concentration, which may inhibit proliferation of some protozoa and zooplankton but not microalgae [55]. On the other hand, significant reduction in oxygen concentration by stopping culture mixing may create an anaerobic environment that may cause deterioration of the culture and thus reduction in productivity [56]. In this study, stopping mixing the culture at night reduced biomass production by 13% and lowered EPA yield by 22%, as compared to the cultures with continuous mixing. The exact reason for the reduction in biomass and EPA yields may deserve further study.

*T. aequale* produced over 30% more biomass in M-Zarrouk culture medium than in BG11 medium (Figure 1). However, the total fatty acid content of the cells in the M-Zarrouk cultures was just roughly one-third of that obtained from the BG11 cultures. Therefore, M-Zarrouk culture medium offered no significant advantage over the BG11-based culture media. Compared to the standard BG11, the additional 0.13 g L^−1^ MgSO_4_ alone or in combination with the additional 0.6 g L^−1^ K_2_HPO_4_ in the BG11 medium further increased the EPA content in the cells. The biomass productivity of 16.2 ± 1.3 g m^−2^ d^−1^ obtained from the mass culture of *T. aequale* in ORP was comparable to that from the culture of another Tribonema species *T. minus* that achieved a biomass productivity of 15.9 ± 0.3 g m^−2^ d^−1^ in 3.5 m^2^ raceway ponds [30]. It was also similar to that from the mass culture of the commercially more popular filamentous cyanobacterium *Arthrospira platensis* in raceway ponds in Ordos, Inner Mongolia (China), that are at roughly the same latitude (YanJiao, Hebei province: 39°96′ N, 116°82′ E, Ordos, Inner Mongolia: 38°18′–40°11′ N, 106°41′–108°54′ E) during the same time of the year [57]. The areal EPA productivity of 542.5 mg m^−2^ d^−1^ obtained from *T. aequale* culture was among the highest figures reported for microalgae-based EPA production in an ORP setting, but somewhat lower than that (i.e., EPA productivity of 650 mg m^−2^ d^−1^) obtained from the cultivation of *Nannochloropsis* sp. in a 500 L flat panel photobioreactor (Table 7). Our results together with previous studies on *Tribonema* spp. [19,20,30,39,58] suggest that *T. aequale* can be an emerging filamentous microalgal species for commercial production of EPA.

**Table 7:** Comparison of *T. aequale* and several other unicellular EPA-producing microalgae in terms of typical biomass concentration and productivity, and the content and productivity of biomass and EPA under laboratory and outdoor environmental conditions.

Species/Strain	Reactors	Operations	Biomass Concentration or Productivity	EPA Content or Percentage of TFA	EPA Yield or Productivity	Ref.
Bacillariophyceae						
*P. tricomutum* PTN0301	70 L PBRs	Indoor, supply with waste CO_2_	−0.7 g L^−1^	24 % TFA	−1.39 mg L^−1^	[59]
*P. tricomutum* CCFM 06	35 L Green Wall Panels	Outdoor, continuous mode, controlled temperature	0.18–0.21 g L^−1^ d^−1^	3.1–4.4% DW	5.7–9.8 mg L^−1^ d^−1^	[60]
*P. tricomutum* UTEX 640	51 L tubular PBRs	Outdoor, fed-batch	1.0 g L^−1^	32.4% TFA	160 mg L^−1^	[61]
*P. tricomutum* UTEX 640	0.785 m^2^ open circular ponds	Outdoor, fed-batch	0.9 g L^−1^	32.6 TFA	60 mg L^−1^	[61]
*Halamphora coffeaeformis*	300 L raceway ponds	Outdoor, batch	0.43 g L^−1^	24 TFA	30 mg L^−1^	[9]
Eustigmatophyceae						
*N. oceanica* CY2	10 L PBRs with immersed lights	Indoor, LED illumination, semi-batch	0.26–0.31 g L^−1^ d^−1^	4.10–4.98% DW	12.6–14.4 mg L^−1^ d^−1^	[62]
*N. oceanica* CY2	5 L plastic bag-type PBRs	Indoor, batch	0.318 g L^−1^ d^−1^	4.14% DW	9.93 mg L^−1^ d^−1^	[63]
*N. gaditana* B-3	216 L flat-panel PBR	Outdoor, continuous	0.13–0.20 g L^−1^ d^−1^	2.08–4.27% DW	4.19–4.85 mg L^−1^ d^−1^	[51]
*N. gaditana*	100 L annular tubular PBRs	Outdoor, semi-continuous	0.078–0.105 g L^−1^ d^−1^	18.76–41.56 TFA	2.4–4.1 mg L^−1^ d^−1^	[64]
*Nannochloropsis* sp.	500 L flat plate glass PBRs	Outdoor, continuous	6.7–12.6 g m^−2^ d^−1^	-	650 mg m^−2^ d^−1^	[65]
*N. salina* CCMP 1776	50 m^2^ raceway ponds	Outdoor, gravity-driven flow	3.47 g m^−2^ d^−1^	−2.8% DW	−97.2 mg m^−2^ d^−1^	[66]
Chrysophyceae						
*Isochrysis galbana* ALII-4	50 L tubular PBRs	Outdoor, semi-continuous	0.28–0.32 g L^−1^ d^−1^	2.56% DW	8.2 mg L^−1^ d^−1^	[67]
Xanthophyceae						
*T. minus*	1.2 L column PBRs	Indoor, cultivation with 100% tofu wastewater	7.7 g L^−1^	1.33% DW	5.73 mg L^−1^ d^−1^	[68]
*T. minus*	3.5 m^2^ raceway ponds	fed by municipal wastewater	15.9 g m^−2^ d^−1^	4.0% DW	−636.00 mg m^−2^ d^−1^	[30]
*T. aequale* SAG200.80	0.9 L column PBRs	Indoor, batch	0.78 g L^−1^ d^−1^	2.9% DW/15.1% TFA	284 mg L^−1^/22.6 mg L^−1^ d^−1^	This study
*T. aequale* SAG200.80	Paddle-driven raceway ponds	Outdoor, batch	39.8–87.5 mg L^−1^ d^−1^/8.6–16.2 g m^−2^ d^−1^	3.1–3.5% DW/25.7–27.5% TFA	1.4–2.7 mg L^−1^ d^−1^/266.9–542.5 mg m^−2^ d^−1^	This study

## 4. Materials and Methods

### 4.1. Organism and Stock Culture Conditions

The filamentous yellow-green microalga *T. aequale* SAG200.80 was obtained from the Culture Collection of Algae at Göttingen University, Germany. The strain was maintained in the BG11 culture medium in 250 mL flasks at a constant temperature of 25 ± 1 °C under continuous illumination of 80 μmol photons m^−2^ s^−1^. The flask cultures were hand-shaken twice a day.

### 4.2. Cultivation of T. aequale SAG200.80 in a Glass Column Photobioreactor

The glass column photobioreactor consisted of 12 glass columns each measuring 4.3 cm inner diameter and 900 mL culture volume. Cultures were maintained at 25 °C with continuous illumination with cool white fluorescence light at a light intensity of 200 μmol m^−2^ s^−1^. Cultures were aerated with compressed air containing 1~2% CO_2_ to maintain a culture pH of 7.5–8.0.

### 4.3. Cultivation of T. aequale SAG200.80 in Open Raceway Ponds (ORP) of Various Sizes under Outdoor Conditions

**Figure 8:**
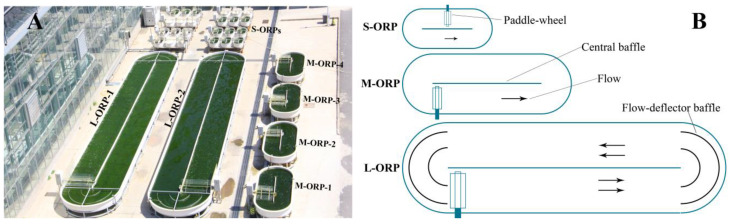
Photograph of ORP of the three sizes (**A**), and schematic diagram of three-size ORP (**B**). The illuminated surface areas of S-ORP, M-ORP, and L-ORP were 0.56, 5.2, and 52 m^2^, respectively.

Cultivation of *T. aequale* SAG200.80 in ORP outdoors was carried out at the SDIC Microalgal Biotechnology Center Testbed facility (Yanjiao, China; 39°96′ N, 116°82′ E) from July through September 2019. Three sizes of ORPs were employed, and they were small-size ORP (S-ORP) with an illuminated surface area of 0.5652 m^2^, medium-size ORP measuring a surface area of 5.2 m^2^, and large-size ORP of 52 m^2^ (Figure 8). A general procedure of preparation of microalgal inoculum, a *Tribonema* culture in glass columns, was scaled up to a 12 L flat panel photobioreactor, then to a 380 L tubular photobioreactor, and finally to a 1500 L tubular photobioreactor.

All the culture experiments started with *Tribonema* filaments obtained at the logarithmic growth phase. Different culture depths, i.e., 10, 15, 20, and 25 cm in ORP were assessed in terms of algal growth and contents and yields of EPA, PLA, and TFA. Algal culture was circulated in an ORP by a paddle wheel at a linear flow rate of 22 ± 2 cm s^−1^. A stable culture pH value of 7.5–8.0 was maintained by fine-bubbling of pure CO_2_ into the cultures during the daylight period. The supply of CO_2_ was halted at night in S-ORP while supplied continuously in larger ponds. The daily evaporation loss in each ORP was compensated by adding tap water. The solar intensity and ambient temperature were recorded by an on-site meteorological station. Culture pH and temperature were measured by a portable pH meter (S2-Meter, Mettler Toledo, Greifensee, Switzerland).

### 4.4. Analytical Methods

#### 4.4.1. Cell Dry Weight Measurement

Biomass concentration was measured in terms of cell dry weight (DW). A certain volume of culture (v) was filtered through a pre-weighed 0.45 μm cellulose acetate membrane filter (JinTeng, Tianjin, China, DW_0_), then washed twice with distilled water and dried at 85 °C overnight, and then weighed (DW_1_). Algal biomass concentration of culture was calculated as Equation (1):

(1) $$\mathrm{DW}=\frac{{\mathrm{DW}}_{1}-{\mathrm{DW}}_{0}}{v}$$

#### 4.4.2. Light Microscopic Observation of T. aequale SAG200.80

Morphological characteristics of *T. aequale* SAG200.80 were observed under microscope. To visualize subcellular lipid bodies, a fluorescence dye, Nile Red (9-diethylamino-5*H*-benzo[a]phenoxazine-5-one; Sigma-Aldrich, St. Louis, MO, USA), was used to stain the organism [69]. Briefly, the Nile Red staining solution was prepared in dimethyl sulfoxide (DMSO) solvent (1/1000, *w*/*v*). A small volume (e.g., 5 μL) of the Nile Red staining solution was added to 1 mL of culture sample with appropriate dilution and incubated in a 45 °C water bath for five minutes. The sample was then cooled down to room temperature (ca. 25 °C), and then another 5 μL of the Nile Red staining solution was added and kept in the water bath for another five minutes. The cells stained with Nile Red were observed and photographed with a fluorescent microscope (BX53, Olympus, Toyko, Japan) equipped with a 100/1.40 oil immersion objective and fluorescence light source components (U-HGLGPS). The excitation wavelength ranged from 505 to 566 nm.

#### 4.4.3. Quantification of Total Fatty Acids

Fatty acid profiles of the organism were determined by a protocol described by Van Wychen, et al. [70] with modifications. In brief, freeze-dried microalgal biomass (10 mg) was added into a glass vial (Agilent Technologies, Santa Clara, CA, USA) containing 300 μL hydrochloric acid and methanol mixtures (5%, *v*/*v*), 200 μL chloroform, and methanol (2:1, *v*/*v*) solutions in the presence of 25 μL of tridecanoic acid (10 mg ml^−1^). Tridecanoic acid, an odd-chain fatty acid that does not naturally occur in microalgae, was transesterified together with the sample to quantify total fatty acid methyl esters (FAMEs) by gas chromatography (GC, Agilent Technologies, Santa Clara, CA, USA) and was used as an internal standard. Extraction and transesterification of fatty acids took place at 85°C for 1 h. The FAMEs were analyzed by an Agilent 7890B + 5977A GC-MS (Agilent Technologies Inc., Santa Clara, CA, USA). The capillary column was HP-88 (60 m × 0.25 mm × 0.2 μm). The initial temperature of the oven was 50 °C and maintained for 2 min, heated at a rate of 25 °C min^−1^ to 175 °C, and maintained for 5 min, then heated again at a rate of 7 °C min^−1^ to 210 °C and maintained for 1 min. The injector temperature was kept at 250 °C in split (20:1) mode for an injection volume of 1 μL. The auxiliary heater, electron ionization (EI) source, and MS Quadrupole temperatures were 250 °C, 230 °C, and 150 °C, respectively. Helium was used as the carrying gas at a flow rate of 1 mL min^−1^.

#### 4.4.4. Identification of Microzooplankton Contaminants

Observations and photomicrography of protozoa and rotifers were made with a differential interference contrast microscope (Olympus microscope BX53, Japan). Species identifications and classification were based on the morphology of the organisms.

### 4.5. Calculations

The EPA, PLA, or TFA content (% DW) were calculated according to Equation (2):

(2) $$\mathrm{EPA}/\mathrm{PLA}/\mathrm{TFA}\;\mathrm{content}=\frac{\mathrm{EPA}/\mathrm{PLA}/\mathrm{TFA}\;\mathrm{weight}}{\mathrm{DW}}\times 100\%$$

Volumetric biomass, EPA, PLA, or TFA productivity (*VP_biomass_* or *VP_EPA/PLA/TFA_*) were calculated as Equations (3) and (4).

(3) $${VP}_{biomass}=\frac{W_{f}-W_{i}}{T}$$

(4) $${VP}_{EPA/PLA/TFA}={VP}_{biomass}\;\times \;\mathrm{EPA}/\mathrm{PLA}/\mathrm{TFA}\;\mathrm{content}$$

where *W_f_* and *W_i_* represented the final and the initial biomass concentration in the culture, respectively, and T was the cultivation time.

The areal biomass productivity (*AP_biomass_*), and productivity of EPA, PLA, or TFA (*AP_EPA/PLA/TFA_*) of cultures in ORP were calculated according to Vadlamani et al. [71] with minor modifications (Equations (5) and (6)).

(5) $${AP}_{biomass}={VP}_{biomass}\;\times \;\frac{V}{A}$$

(6) $${AP}_{EPA/PLA/TFA}={VP}_{EPA/PLA/TFA}\;\times \;\frac{V}{A}$$

where V is the culture volume and A is the surface area of ORP.

## 5. Conclusions

The yellow-green filamentous microalga *T. aequale* cells contained 2.9% of EPA (*w*/*w*) and reached a biomass concentration of 9.8 g L^−1^ in a glass column photobioreactor under laboratory conditions. The very high cellular EPA content of 3.5%, and the high areal biomass and EPA productivities of 16.2 g m^−2^ d^−1^ and 542.5 mg m^−2^ d^−1^, respectively, were obtained from an outdoor open raceway pond study. The high yield potential of biomass and EPA production and high resistance to microbial grazers as well as easy biomass harvesting could make *T. aequale* an ideal organism for commercial EPA production.

## Figures and Tables

**Table 1:** Nutrient compositions of the culture media used in the study; ns means the chemical compositions of A5 solution are not shown.

Nutrients	Medium and Concentration (g L^−1^)				
	BG11	M-Zarrouk	BG11 + P	BG11 + Mg	BG11 + P + Mg
CO(NH_2_)_2_	0.54	0.54	0.54	0.54	0.54
K_2_HPO_4_·3H_2_O	0.0524	0.655	0.655	0.0524	0.655
Na_2_CO_3_	0.02	-	0.02	0.02	0.02
MgSO4·7H_2_O	0.075	0.2	0.075	0.2	0.2
CaCl_2_	0.0272	0.04	0.0272	0.0272	0.0272
Citric acid	0.006	-	0.006	0.006	0.006
Ammonium ferric citrate	0.006	-	0.006	0.006	0.006
Na_2_EDTA	0.001	0.08	0.001	0.001	0.001
FeSO_4_·7H_2_O	-	0.01	-	-	-
A5 solution	ns	ns	ns	ns	ns

**Table 2:** The final biomass concentration, the cellular EPA, PLA, and TFA contents, and productivities of biomass, EPA, PLA, and TFA in *T. aequale* cultures in 0.56 m^2^-ORP. The values from the experiments (*n* = 3) are shown as mean ± one standard deviation. The different lowercase letters indicate statistically significant differences.

Medium	Final Biomass Concentration (g L^−1^)	Areal Biomass Productivity (g m^−2^ d^−1^)	EPA Content (%)	Areal EPA Productivity (g m^−2^ d^−1^)	PLA Content (%)	Areal PLA Productivity (g m^−2^ d^−1^)	TFA Content (%)	Areal TFA Productivity (g m^−2^ d^−1^)
1/2BG11	1.10 ± 0.03	15.5 ± 0.73 ^ab^	3.50 ± 0.01	542.5 ± 27.1 ^a^	3.68 ± 0.05	570.4 ± 34.6 ^a^	11.19 ± 0.11	1734.5 ± 98.7 ^a^
BG11	1.08 ± 0.01	15.3 ± 0.42 ^b^	3.25 ± 0.01	495.6 ± 15.2 ^b^	3.45 ± 0.02	526.1 ± 17.5 ^b^	10.66 ± 0.05	1625.7 ± 52.4 ^a^
BG11 + P + Mg	1.15 ± 0.05	16.2 ± 1.26 ^a^	3.07 ± 0.03	496.4 ± 43.5 ^b^	3.27 ± 0.03	528.8 ± 46.1 ^b^	10.39 ± 0.09	1680.1 ± 145.5 ^a^

**Table 3:** Fatty acid profiles (% TFA) of *T. aequale* cultured with the different media: 1/2 BG11, BG11, and BG11 + P + Mg. Values are expressed as mean ± standard deviation of two replicates. In BG11 + P + Mg culture medium were the additional amounts of P and Mg, i.e., of 0.60 g L^−1^ K_2_HPO_4_ and 0.13 g L^−1^ MgSO_4_.

Fatty Acid Group	1/2BG11	BG11	BG11 + P + Mg
C14:0	2.77 ± 0.02	2.66 ± 0.01	2.70 ± 0.01
C16:0	12.53 ± 0.01	13.36 ± 0.05	13.89 ± 0.12
C16:1	32.87 ± 0.08	32.26 ± 0.14	31.47 ± 0.04
C16:2	4.74 ± 0.08	4.96 ± 0.04	5.29 ± 0.02
C16:3	4.19 ± 0.02	4.71 ± 0.00	5.17 ± 0.01
C18:0	0.19 ± 0.00	0.18 ± 0.01	0.20 ± 0.04
C18:1	0.38 ± 0.02	0.33 ± 0.00	0.31 ± 0.00
C18:2	0.90 ± 0.03	0.92 ± 0.05	1.14 ± 0.03
C18:3	1.24 ± 0.01	1.27 ± 0.00	1.32 ± 0.01
C20:3	0.29 ± 0.03	0.28 ± 0.01	0.29 ± 0.02
C20:4	8.63 ± 0.07	8.47 ± 0.02	8.67 ± 0.06
C20:5	31.26 ± 0.36	30.50 ± 0.06	29.55 ± 0.06
∑SFA	15.49 ± 0.03	16.20 ± 0.07	16.79 ± 0.17
∑MUFA	33.25 ± 0.10	32.59 ± 0.14	31.78 ± 0.13
∑PUFA	51.25 ± 0.68	51.11 ± 0.18	51.43 ± 0.21

**Table 4:** EPA, PLA, and TFA contents, proportions of EPA and PLA in TFA, and areal productivities of EPA, PLA, and TFA as affected by culture depth ranging from 10 to 25 cm. The values from the experiments (*n* = 3) are shown as mean ± one standard deviation. The lowercase letters indicate statistically significant differences.

Culture Depth	EPA Content (% DW)	EPA Proportion (% TFA)	PLA Content (% DW)	PLA Proportion (% TFA)	TFA Content (% DW)	*AP_EPA_* (mg m^−2^·d^−1^)	*AP_PLA_* (mg m^−2^·d^−1^)	*AP_TFA_* (mg m^−2^·d^−1^)
10 cm	3.05 ± 0.02 ^c^	26.34 ± 0.12 ^b^	4.09 ± 0.05 ^b^	35.38 ± 0.07 ^a^	11.56 ± 0.11 ^c^	266.89 ± 3.51 ^c^	357.88 ± 6.74 ^c^	1011.50 ± 16.30 ^c^
15 cm	3.15 ± 0.03 ^b^	25.70 ± 0.37 ^b^	4.27 ± 0.01 ^a^	34.84 ± 0.09 ^b^	12.27 ± 0.08 ^b^	313.03 ± 7.95 ^b^	424.33 ± 8.75 ^b^	1219.33 ± 27.86 ^b^
20 cm	3.39 ± 0.02 ^a^	27.00 ± 0.01 ^ab^	4.36 ± 0.01 ^a^	34.79 ± 0.05 ^b^	12.53 ± 0.04 ^a^	349.17 ± 10.00 ^a^	449.08 ± 12.05 ^a^	1290.59 ± 35.21 ^a^
25 cm	3.42 ± 0.01 ^a^	27.51 ± 0.06 ^a^	4.27 ± 0.01 ^a^	34.47 ± 0.08 ^c^	12.40 ± 0.05 ^ab^	339.86 ± 7.45 ^b^	424.33 ± 9.20 ^b^	1232.25 ± 27.15 ^ab^

**Table 5:** EPA, PLA, and TFA contents, proportions of EPA and PLA in TFA, and areal productivities of EPA, PLA, and TFA of *T. aequale* grown in 5.2 m^2^ M-ORP operated in a continuous mixing mode and a daytime mixing one. The values from the experiments (*n* = 2) are shown as mean.

Mixing Regimes	EPA Content (% DW)	PLA Content (% DW)	TFA Content (% DW)	EPA Proportion (% TFA)	PLA Proportion (% TFA)	*AP_EPA_* (mg m^−2^ d^−1^)	*AP_PLA_* (mg m^−2^ d^−1^)	*AP_TFA_* (mg m^−2^ d^−1^)
Continuous mixing	3.23	4.50	11.89	27.13	37.92	370.84	518.52	1367.19
Daytime mixing	3.02	3.81	10.65	28.57	35.76	289.01	361.69	1011.57

**Table 6:** EPA, PLA, and TFA contents, proportions of EPA and PLA in TFA, and areal productivities of EPA, PLA, and TFA of *T. aequale* grown in two 52 m^2^ L-ORP outdoors operated in a continuous culture mixing mode.

L-ORP	EPA Content (% DW)	PLA Content (% DW)	TFA Content (% DW)	EPA Proportion (% TFA)	PLA Proportion (% TFA)	*AP_EPA_* (mg m^−2^ d^−1^)	*AP_PLA_* (mg m^−2^ d^−1^)	*AP_TFA_* (mg m^−2^ d^−1^)
L-ORP-1	3.20	3.96	11.69	27.38	33.85	343.42	424.60	1254.45
L-ORP-2	3.25	3.93	11.64	28.00	33.74	447.19	538.89	1597.06

## Data Availability

The data presented in this study are available in this article.

## References

[1] Wang Y., Li R., Hildebrand D.F. (2012). Biosynthesis and metabolic engineering of palmitoleate production, an important contributor to human health and sustainable industry. Prog. Lipid Res..

[2] Bellou S., Triantaphyllidou I.E., Aggeli D., Elazzazy A.M., Baeshen M.N., Aggelis G. (2016). Microbial oils as food additives: Recent approaches for improving microbial oil production and its polyunsaturated fatty acid content. Curr. Opin. Biotechnol..

[3] Qi B., Fraser T., Mugford S., Dobson G., Sayanova O., Butler J., Napier J.A., Stobart A.K., Lazarus C.M. (2004). Production of very long chain polyunsaturated omega-3 and omega-6 fatty acids in plants. Nat. Biotechnol..

[4] Petrie J.R., Shrestha P., Belide S., Kennedy Y., Lester G., Liu Q., Divi U.K., Mulder R.J., Mansour M.P., Nichols P.D. (2014). Metabolic engineering *Camelina sativa* with fish oil-like levels of DHA. PLoS ONE.

[5] Sirisuk P., Ra C.H., Jeong G.T., Kim S.K. (2018). Effects of wavelength mixing ratio and photoperiod on microalgal biomass and lipid production in a two-phase culture system using LED illumination. Bioresour. Technol..

[6] Cook O., Hildebrand M. (2016). Enhancing LC-PUFA production in *Thalassiosira pseudonana* by overexpressing the endogenous fatty acid elongase genes. J. Appl. Phycol..

[7] Han D., Jia J., Li J., Sommerfeld M., Xu J., Hu Q. (2017). Metabolic remodeling of membrane glycerolipids in the microalga *Nannochloropsis oceanica* under nitrogen deprivation. Front. Mar. Sci..

[8] Yang R., Wei D., Xie J. (2020). Diatoms as cell factories for high-value products: Chrysolaminarin, eicosapentaenoic acid, and fucoxanthin. Crit. Rev. Biotechnol..

[9] Popovich C.A., Faraoni M.B., Sequeira A., Daglio Y., Martín L.A., Martínez A.M., Damiani M.C., Matulewicz M.C., Leonardi P.I. (2020). Potential of the marine diatom *Halamphora coffeaeformis* to simultaneously produce omega-3 fatty acids, chrysolaminarin and fucoxanthin in a raceway pond. Algal Res..

[10] Lu L., Wang J., Yang G., Zhu B., Pan K. (2017). Heterotrophic growth and nutrient productivities of *Tetraselmis chuii* using glucose as a carbon source under different C/N ratios. J. Appl. Phycol..

[11] Wen Z.Y., Chen F. (2000). Production potential of eicosapentaenoic acid by the diatom *Nitzschia laevis*. Biotechnol. Lett..

[12] Hu Q., Hu Z., Cohen Z., Richmond A. (1997). Enhancement of eicosapentaenoic acid (EPA) and Gamma-linolenic acid (GLA) production by manipulating algal density of outdoor cultures of *Monodus subterraneus* (Eustigmatophyta) and *Spirulina platensis* (Cyanobacteria). Eur. J. Phycol..

[13] Song M., Pei H., Hu W., Ma G. (2013). Evaluation of the potential of 10 microalgal strains for biodiesel production. Bioresour. Technol..

[14] Wang F., Gao B., Huang L., Su M., Dai C., Zhang C. (2018). Evaluation of oleaginous Eustigmatophycean microalgae as potential biorefinery feedstock for the production of palmitoleic acid and biodiesel. Bioresour. Technol..

[15] Matsunaga T., Takeyama H., Miura Y., Yamazaki T., Furuya H., Sode K. (1995). Screening of marine cyanobacteria for high palmitoleic acid production. FEMS Microbiol. Lett..

[16] Zhang A., Wen X., Wang K., Huo Y., Geng Y., Ding Y., Li Y. (2021). Using surfactants for controlling rotifer contamination in mass cultivation of *Chlorella pyrenoidosa*. Algal Res..

[17] Park S.H., Steichen S.A., Li X., Ogden K., Brown J.K. (2019). Association of *Vampirovibrio chlorellavorus* with decline and death of *Chlorella sorokiniana* in outdoor reactors. J. Appl. Phycol..

[18] Wang H., Gao L., Chen L., Guo F., Liu T. (2013). Integration process of biodiesel production from filamentous oleaginous microalgae *Tribonema minus*. Bioresour. Technol..

[19] Wang H., Gao L., Zhou W., Liu T. (2016). Growth and palmitoleic acid accumulation of filamentous oleaginous microalgae *Tribonema minus* at varying temperatures and light regimes. Bioprocess. Biosyst. Eng..

[20] Zhou W., Wang H., Zheng L., Cheng W., Gao L., Liu T. (2019). Comparison of lipid and palmitoleic acid induction of *Tribonema minus* under heterotrophic and phototrophic regimes by using high-density fermented seeds. Int. J. Mol. Sci..

[21] Hu H., Li J., Pan X., Zhang F., Ma L., Wang H., Zeng R.J. (2019). Different DHA or EPA production responses to nutrient stress in the marine microalga *Tisochrysis lutea* and the freshwater microalga *Monodus subterraneus*. Sci. Total Environ..

[22] Liang J., Iqbal S., Wen F., Tong M., Liu J. (2019). Phosphorus-induced lipid class alteration revealed by lipidomic and transcriptomic profiling in oleaginous microalga *Nannochloropsis* sp. PJ12. Mar. Drugs.

[23] Rissler H.M., Collakova E., DellaPenna D., Whelan J., Pogson B.J. (2002). Chlorophyll Biosynthesis. Expression of a Second *Chl I* Gene of Magnesium Chelatase in Arabidopsis Supports Only Limited Chlorophyll Synthesis. Plant Physiol..

[24] Valentine R.C., Valentine D.L. (2004). Omega-3 fatty acids in cellular membranes: A unified concept. Prog. Lipid Res..

[25] Muys M., Sui Y., Schwaiger B., Lesueur C., Vandenheuvel D., Vermeir P., Vlaeminck S.E. (2019). High variability in nutritional value and safety of commercially available Chlorella and Spirulina biomass indicates the need for smart production strategies. Bioresour. Technol..

[26] Borowitzka L.J., Borowitzka M.A. (1990). Commercial production of β-carotene by *Dunaliella salina* in open ponds. Bull. Mar. Sci..

[27] Nishshanka G.K.S.H., Liyanaarachchi V.C., Nimarshana P.H.V., Ariyadasa T.U., Chang J.S. (2022). *Haematococcus pluvialis*: A potential feedstock for multiple-product biorefining. J. Clean. Prod..

[28] Ugwu C.U., Aoyagi H., Uchiyama H. (2008). Photobioreactors for mass cultivation of algae. Bioresour. Technol..

[29] Lam M.K., Lee K.T. (2012). Microalgae biofuels: A critical review of issues, problems and the way forward. Biotechnol. Adv..

[30] Davis A.K., Anderson R.S., Spierling R., Leader S., Lesne C., Mahan K., Lundquist T., Benemann J.R., Lane T., Polle J.E. (2021). Characterization of a novel strain of *Tribonema minus* demonstrating high biomass productivity in outdoor raceway ponds. Bioresour. Technol..

[31] Sutherland D.L., Turnbull M.H., Craggs R.J. (2014). Increased pond depth improves algal productivity and nutrient removal in wastewater treatment high rate algal ponds. Water Res..

[32] Sawant S.S., Khadamkar H.P., Mathpati C.S., Pandit R., Lali A.M. (2018). Computational and experimental studies of high depth algal raceway pond photo-bioreactor. Renew. Energy.

[33] Chiaramonti D., Prussi M., Casini D., Tredici M.R., Rodolfi L., Bassi N., Zittelli G.C., Bondioli P. (2013). Review of energy balance in raceway ponds for microalgae cultivation: Re-thinking a traditional system is possible. Appl. Energy.

[34] Vadiveloo A., Shayesteh H., Bahri P.A., Moheimani N.R. (2022). Comparison between continuous and daytime mixing for the treatment of raw anaerobically digested abattoir effluent (ADAE) and microalgae production in open raceway ponds. Bioresour. Technol. Rep..

[35] Cuello M.C., Cosgrove J.J., Randhir A., Vadiveloo A., Moheimani N.R. (2015). Comparison of continuous and day time only mixing on *Tetraselmis suecica* (Chlorophyta) in outdoor raceway ponds. J. Appl. Phycol..

[36] Vadiveloo A., Moheimani N. (2018). Effect of continuous and daytime mixing on *Nannochloropsis* Growth in Raceway Ponds. Algal Res..

[37] Zarrouk C. (1966). Contribution A l’etude d’une Cyanobacterie. Influence de Divers Facteurs Physiques et Chimiques sur la Croissance et la Photosynthese de *Spirulina maxima*. Ph.D. Thesis.

[38] Uduman N., Qi Y., Danquah M.K., Forde G.M., Hoadley A. (2010). Dewatering of microalgal cultures: A major bottleneck to algae-based fuels. J. Renew. Sustain. Energy.

[39] Zhou W., Wang H., Chen L., Cheng W., Liu T. (2017). Heterotrophy of filamentous oleaginous microalgae *Tribonema minus* for potential production of lipid and palmitoleic acid. Bioresour. Technol..

[40] Day J.G., Gong Y., Hu Q. (2017). Microzooplanktonic grazers-A potentially devastating threat to the commercial success of microalgal mass culture. Algal Res..

[41] Gromov B.V. (1972). Apheldium tribonemae Scherffel parasitizing yellow green algae. Mikol. Fitopatol..

[42] Karpov S.A., Mamkaeva M.A., Benzerara K., Moreira D., López-García P. (2014). Molecular phylogeny and ultrastructure of *Aphelidium aff. melosirae* (Aphelida, Opisthosporidia). Protist.

[43] Xu Z., He Q., Gong Y., Wang Y., Chi Q., Liu G., Hu Z., Zhang C., Hu Q. (2021). Assessment of a novel oleaginous filamentous microalgae *Klebsormidium* sp. Lgx80 (Streptophyta, Klebsormidiales) for biomass and lipid production. J. Phycol..

[44] Wang Y., Jia J., Chi Q., Li Y., Wang H., Gong Y., Liu G., Hu Z., Han D., Hu Q. (2022). Critical assessment of the filamentous green microalga *Oedocladium carolinianum* for astaxanthin and oil production. Algal Res..

[45] Christenson L., Sims R. (2011). Production and harvesting of microalgae for wastewater treatment, biofuels, and bioproducts. Biotechnol. Adv..

[46] Eustance E., Wray J.T., Badvipour S., Sommerfeld M.R. (2016). The effects of cultivation depth, areal density, and nutrient level on lipid accumulation of *Scenedesmus acutus* in outdoor raceway ponds. J. Appl. Phycol..

[47] Cunha P., Pereira H., Costa M., Pereira J., Silva J.T., Fernandes N., Varela J., Silva J., Simões M. (2020). *Nannochloropsis oceanica* cultivation in polot-scale raceway ponds—From design to cultivation. Appl. Sci..

[48] Chuka-ogwude D., Nafisi M., Vadiveloo A., Taher H., Bahri P.A., Moheimani N.R. (2021). Effect of medium recycling, culture depth, and mixing duration on *D. salina* growth. Algal Res..

[49] Richmond A., Hu Q. (1997). Principles for efficient utilization of light for mass production of photoautotrophic microorganisms. Biotechnology for Fuels and Chemicals.

[50] Xin Y., Shen C., She Y., Chen H., Wang C., Wei L., Yoon K., Han D., Hu Q., Xu J. (2019). Biosynthesis of triacylglycerol molecules with a tailored PUFA profile in industrial microalgae. Mol. Plant.

[51] Camacho-Rodríguez J., González-Céspedes A.M., Cerón-García M.C., Fernández-Sevilla J.M., Acién-Fernández F.G., Molina-Grima E. (2014). A quantitative study of eicosapentaenoic acid (EPA) production by *Nannochloropsis gaditana* for aquaculture as a function of dilution rate, temperature and average irradiance. Appl. Microbiol. Biotechnol..

[52] Ramus J., Beale S.I., Mauzerall D., Howard K.L. (1976). Changes in photosynthetic pigment concentration in seaweeds as a function of water depth. Mar. Biol..

[53] Pajot A., Hao Huynh G., Picot L., Marchal L., Nicolau E. (2022). Fucoxanthin from algae to human, an extraordinary bioresource: Insights and advances in up and downstream processes. Mar. Drugs.

[54] Rogers J.N., Rosenberg J.N., Guzman B.J., Oh V.H., Mimbela L.E., Ghassemi A., Betenbaugh M.J., Oyler G.A., Donohue M.D. (2014). A critical analysis of paddlewheel-driven raceway ponds for algal biofuel production at commercial scales. Algal Res..

[55] Montemezzani V., Duggan I.C., Hogg I.D., Craggs R.J. (2017). Screening of potential zooplankton control technologies for wastewater treatment high rate algal ponds. Algal Res..

[56] Richmond A. (2013). Biological principles of mass cultivation of photoautotrophic microalgae. Handbook of Microalgal Culture: Applied Phycology and Biotechnology.

[57] Wang X., Jin G., Pan K., Zhu B., Li Y. (2021). Effects of fluctuating temperature in open raceway ponds on the biomass accumulation and harvest efficiency of *Spirulina* in large-scale cultivation. Environ. Sci. Pollut. Res..

[58] Guo F., Wang H., Wang J., Zhou W., Gao L., Chen L., Dong Q., Zhang W., Liu T. (2014). Special biochemical responses to nitrogen deprivation of filamentous oleaginous microalgae *Tribonema* sp. Bioresour. Technol..

[59] Simonazzi M., Pozzolesi L., Guerrini F., Vanucci S., Samorì C., Pistocchi R. (2019). Use of waste carbon dioxide and pre-treated liquid digestate from biogas process for *Phaeodactylum tricornutum* cultivation in photobioreactors and open ponds. Bioresour. Technol..

[60] Steinrücken P., Prestegard S.K., de Vree J.H., Storesund J.E., Pree B., Mjøs S.A., Erga S.R. (2018). Comparing EPA production and fatty acid profiles of three *Phaeodactylum tricornutum* strains under western Norwegian climate conditions. Algal Res..

[61] Benavides A.M.S., Torzillo G., Kopecký J., Masojídek J. (2013). Productivity and biochemical composition of *Phaeodactylum tricornutum* (Bacillariophyceae) cultures grown outdoors in tubular photobioreactors and open ponds. Biomass Bioenergy.

[62] Chen C.Y., Chen Y.C., Huang H.C., Ho S.H., Chang J.S. (2015). Enhancing the production of eicosapentaenoic acid (EPA) from *Nannochloropsis oceanica* CY2 using innovative photobioreactors with optimal light source arrangements. Bioresour. Technol..

[63] Chen C.Y., Nagarajan D., Cheah W.Y. (2018). Eicosapentaenoic acid production from *Nannochloropsis oceanica* CY2 using deep sea water in outdoor plastic-bag type photobioreactors. Bioresour. Technol..

[64] Nogueira N., Nascimento F.J., Cunha C., Cordeiro N. (2020). *Nannochloropsis gaditana* grown outdoors in annular photobioreactors: Operation strategies. Algal Res..

[65] Cheng-Wu Z., Zmora O., Kopel R., Richmond A. (2001). An industrial-size flat plate glass reactor for mass production of *Nannochloropsis* sp. (Eustigmatophyceae). Aquaculture.

[66] Crowe B., Attalah S., Agrawal S., Waller P., Ryan R., Van Wagenen J., Chavis A., Kyndt J., Kacira M., Ogden K.L. (2012). A comparison of *Nannochloropsis salina* growth performance in two outdoor pond designs: Conventional raceways versus the ARID pond with superior temperature management. Int. J. Chem. Eng..

[67] Grima E.M., Pérez J.S., Camacho F.G., Sánchez J.G., Fernández F.A., Alonso D.L. (1994). Outdoor culture of *Isochrysis galbana* ALII-4 in a closed tubular photobioreactor. J. Biotechnol..

[68] Wang F., Gao B., Su M., Dai C., Huang L., Zhang C. (2019). Integrated biorefinery strategy for tofu wastewater biotransformation and biomass valorization with the filamentous microalga *Tribonema minus*. Bioresour. Technol..

[69] Greenspan P., Mayer E.P., Fowler S.D. (1985). Nile red: A selective fluorescent stain for intracellular lipid droplets. J. Cell Biol..

[70] Van Wychen S., Ramirez K., Laurens L.M.L. (2016). Determination of Total Lipids as Fatty Acid Methyl Esters (FAME) by in Situ Transesterification Laboratory Analytical Procedure (LAP).

[71] Vadlamani A., Pendyala B., Viamajala S., Varanasi S. (2019). High productivity cultivation of microalgae without concentrated CO_2_ input. ACS Sustain. Chem. Eng..

